# Successful simultaneous targeting of IgE and IL-5 in a severe asthmatic patient selected for lung transplantation^☆^

**DOI:** 10.1016/j.waojou.2022.100669

**Published:** 2022-07-31

**Authors:** Karl-Christian Bergmann, Jörg-Wilhelm Oestmann, Jean Bousquet, Torsten Zuberbier

**Affiliations:** a Fraunhofer Institute for Translational Medicine and Pharmacology ITMP Allergology and Immunology, Berlin, Germany; b Institute of Allergology, Charité – Universitätsmedizin Berlin, Corporate Member of Freie Universität Berlin and Humboldt-Universität zu Berlin, Berlin, Germany; c Charité *–* Universitätsmedizin Berlin, Corporate Member of Freie Universität Berlin, Humboldt-Universität zu Berlin, Berlin Institute of Health, Berlin, Germany; d University of Montpellier, Montpellier, France; e Centre Hospitalier Universitaire de Montpellier (CHU), Montpellier, France; f Contre les Maladies Chroniques pour un VIeillissement Actif (MACVIA) en France, European Innovation Partnership on Active and Healthy Ageing Reference Site, Montpellier, France; g Department of Dermatology and Allergy Comprehensive Allergy Center Berlin Institute of Health, Berlin, Germany

**Keywords:** Omalizumab, Mepolizumab, Benralizumab, Simultaneous use of biologics, Severe allergic and eosinophilic asthma

## Abstract

We report a case of severe uncontrolled allergic and eosinophilic asthma in which omalizumab had led to a fast remission. After 18 months, mepolizumab was added to omalizumab because of increased blood eosinophils and a deterioration of asthma control. Asthma was then under control for the next 18 months. Discontinuation of mepolizumab in the ensuing 6 months led to a decrease in asthma control and an increased eosinophilia. The introduction of benralizumab resulted in an immediate increase of lung function, asthma control test (ACT), and symptom relief. Before the introduction of biologics, the patient was on the list for transplantation due to respiratory insufficiency. High-resolution CT scans before and after biologic therapy demonstrated a reduction of bronchial wall thickening and mucous plugging as well as an increase in bronchial caliber. The patient did therefore not need a transplant. We conclude that the dual use of biologics may be efficient in some cases of severe asthma.

A man (born in 1969) had suffered from allergic rhinitis to pollen and animal dander since childhood. Persistent asthma occurred in 1993 and became severe in 2007. Despite very good adherence, multiple visits to a disease management program, continuous therapy with high doses of inhaled steroid, long-acting beta-agonists, tiotropium, oral steroids, long-term oxygen therapy, and inpatient rehabilitation, the patient was admitted to the intensive care unit (ICU) in 2010 and 2011. Nasal polyps had been operated on 3 times. The use of aspirin and ibuprofen had caused very severe dyspnea. The patient became unable to work and reported to the Charité Transplant Centre in Berlin. Because of bronchiectasis and respiratory insufficiency despite constant inhalation of oxygen, the Charité Transplant Centre put him on the list of transplant candidates.

A chest CT in July 2021 demonstrated severe bronchiectasis, predominantly in both lower lung lobes.

On presentation at our Centre in July 2017, we saw a nonsmoker, 177 cm tall, 83 kg body weight, with severe orthopnea despite oxygen (3 L/min), 10 mg prednisolone daily, high dose of inhaled steroid (1000 μg), long-acting beta-agonist (salmeterol 100 μg/day), and tiotropium (36 μg/day).

The diagnosis allergic asthma was supported by a positive family history, the occurrence of allergic rhinoconjunctivitis caused by pollen and animal dander (cats, dogs) and the subsequent occurrence of asthmatic symptoms since early childhood. Multiple allergy tests revealed IgE-specific antibodies in the blood against pollen, animal dander and house dust mites. A positive methacholine test (2002) confirmed the bronchial hyperreactivity. Eosinophilia was low with long-term oral corticosteroid (OCS) medication. Interstitial lung disease was ruled out by multiple computed tomography scans of the thorax. Pulmonary hypertension was excluded. The diffusion capacity is almost normal. Bronchial tree abnormalities could not be seen on bronchoscopy. Neuromuscular diseases were ruled out by genetic tests, as was cystic fibrosis as early as childhood.

Functional data were as follows: FEV1 of 1.0 L = 27% (52% FVC), TLC 6.1 L (87%), diffusion capacity 88%, heart rate 115/min, oxygen saturation 95%, ACT 7. IgE 893 kU/L, specific IgE antibody positive to dog, cat, birch and grass pollen, and house dust mites. Eosinophils 120/μl (under 10 mg OCS), lymphopenia (11.4%). Severe exertional dyspnea (7 steps possible).

Symptoms were as follows: Wheeze, dry cough, night waking 5–7 times/week, intense bronchial hyperreactivity. Comorbidities included arterial hypertension and OCS-induced osteoporosis.

Our therapy over the past 4 years has consisted of 4 phases, which have illustrated the effect of omalizumab alone and the additional effect of a second anti-Il-5 biologic.

## Phase 1: Use of omalizumab

On 9 September 2017, omalizumab s.c. (450 mg every two weeks) was started with otherwise unchanged medication. The result was a rapid and dramatic increase in FEV1 and peak flow and an improvement of symptoms. Prednisolone was reduced to 5 mg after 2 months and discontinued after 7 months. Theophylline was able to be discontinued after 6 weeks, and SABA reduced to 2–3 puffs/day. In February 2018 and August 2018, respiratory infections induced asthma exacerbations.

## Phase 2: Use of omalizumab and mepolizumab

On 27 February 2019, mepolizumab 30 mg/4 weeks was started (with informed consent) due to eosinophilia (300/μl in the blood). An immediate rapid reduction of symptoms and improvement in lung function ensued to levels better than those with omalizumab therapy alone. The situation remained stable for 18 months with constant good functional values. The patient was under good control.

## Phase 3: Omalizumab only

Mepolizumab was withdrawn on 16 July 2020 (with the patient's agreement) in order to clarify the necessity of the second biologic.

After discontinuing mepolizumab while maintaining omalizumab, symptoms increased in the second month after the last mepolizumab injection. Furthermore, lung function deteriorated to the same level as in the omalizumab-alone phase in 2018. On 27 January 2021, an eosinophilia (430/μl) was found in blood.

## Phase 4: Omalizumab and benralizumab

Further to the failure of the withdrawal attempt, we started on 27 January 2021 with benralizumab (30 mg 3 times with intervals of 4 weeks, thereafter every 8 weeks) instead of mepolizumab to take advantage of the longer injection intervals compared to mepolizumab by very similar actions of the 2 biologics. The pen injections were administered 2 weeks after the omalizumab injections. Again, a rapid increase in lung function and improvement in symptoms for the next 7 months was seen (latest clinical check on 24.11.2021).

**Fig. 1:**
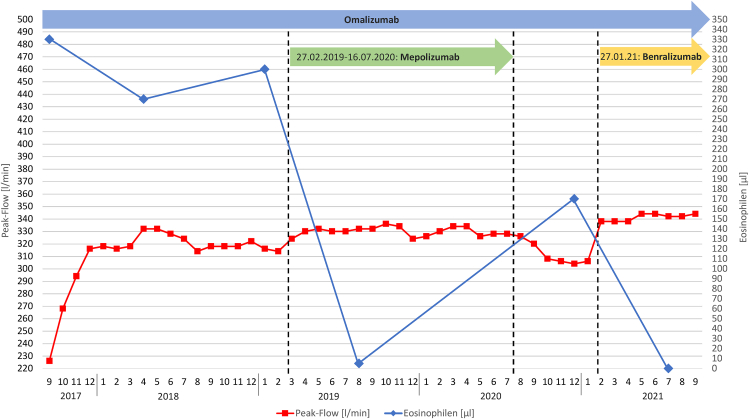
Course of the peak flow values from September 2017 to September 2021, mean monthly values of the daily morning values and number of eosinophils

The summarizing figure shows the development of peak-flow values since the start of omalizumab in September 2021 (see Fig. 1).

## Chest high-resolution CT

**Fig. 2:**
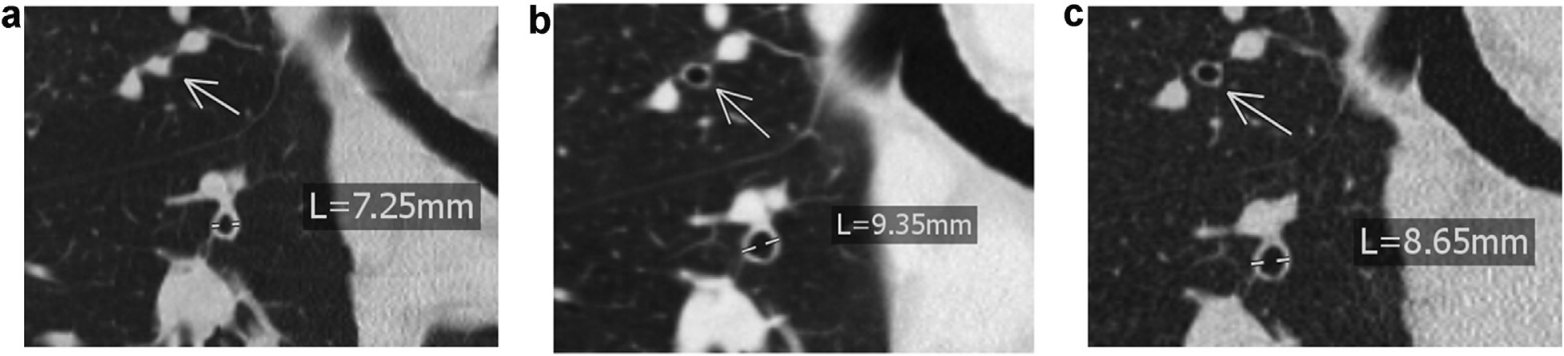
Comparison of CT findings in 2017 (a), January 2021 (b), and September 2021 (c) shows a decrease of mucous plugging (arrow) and bronchial wall thickening as well as an increase in bronchial caliber

High resolution CT scans of the lung were taken before biologic therapy in July 2017, at the beginning of omalizumab and benralizumab in January 2021, and in September 2021 to check for possible changes. We found a decrease of mucous plugging and bronchial wall thickening as well as an increase in bronchial caliber (Fig. 2), supporting the improved lung function and reduced symptoms.

## Clinical impact of the therapy

The clinical impact of the therapy of omalizumab alone was already very impressive and under the simultaneous use of the 2 biologics even higher. The use of OCS could be discontinued, as could long-term oxygen therapy, The 6-minute walking distance (under 4 L of oxygen/min) was extended from 360 m to an unlimited distance without oxygen. Before biological therapy, the patient was practically unable to leave the house alone, but was able to fly to Mallorca with his family in summer 2019. The ACT rose from 6 points to 20 points after one year. Exacerbations did not occur with the biologics. The quality of life has been restored.

## Discussion

This clinical case is of interest since it shows that combining biologics in asthma can be beneficial in the most severe patients. It has another important impact since an asthmatic patient who was proposed a lung transplant recovered using biologics.

The phenotyping of severe asthma allows the precise use of biologics. For severe allergic asthma: omalizumab,^1^ for eosinophilic asthma: anti-IL5/anti-IL5 receptor treatment,^2^, ^3^, ^4^ and for asthma with type 2 inflammation: dupilumab.^5^

Here we report on a man with very severe asthma who has (i) a typical allergic background: allergic rhinitis to pollen, animal hair, and house dust mites since childhood, (ii) high IgE, (iii) specific IgE antibodies against seasonal and perennial allergens, (iv) non-allergic mechanisms, and (v) aspirin-exacerbated respiratory disease (AERD). Although dual biologic therapy is not uncommon in severe asthma, it is rare.^6^ In AERD, many patients try many different biologics^7^^,^^8^ as is the case in this report.

Besides severe asthma, the patient was suffering from severe bronchiectasis (shown using a CT-scan) and a resulting chronic respiratory failure. After intense diagnostic and multiple consultations, the Charité Transplantation Centre concluded that a lung transplant could be an option for the patient, but proposed first of all to set up a consultation in our Centre for severe asthma. Interestingly, biologics improved asthma control and reduced bronchiectasis. It is clear that bronchiectasis is relatively common in asthma^9^ and that bronchiectasis is associated with severe asthma.^10^ However, this important finding suggests that (i) bronchiectasis demonstrated by CT-scan may be associated with asthma mechanisms and reversed by biologics and (ii) patients with severe asthma requiring a transplantation may be first treated using biologics.

In individual cases in which 1 biologic does not have a safe effect, switching from one biologic to another can be helpful and successful.^11^

What would have happened if the patient had received a lung transplant? Would he have been free of asthma? It has been reported that asthmatic recipients of normal lungs do not suffer from asthma up to 3 years after transplant.^12^ That would have been a good option.

In contrast, it also has been reported that asthmatic donor lungs have transferred the disease to 2 non-asthmatic recipients who after a very short period complained of asthmatic symptoms.^13^ These observations would support the notion that asthma is a “local” disease. But a case that asthma developed in a lung transplant patient 11 years post-transplant has also been reported.^14^ In summary, the situation looks confusing and for the patient the use of biologicals was the best solution.

## Abbreviations

ACT, asthma control test; AERD, aspirin-exacerbated respiratory disease; CT, computer tomography; FEV1, forced expiratory volume in the first second; FVC, forced vital capacity; ICU, intensive care unit; IgE, Immunoglobulin E; IL-5, interleukin 5; OCS, oral corticoidsteroids; PEF, peak expiratory flow; SABA, short-acting beta-agonist; TLC, total lung capacity.

## Consent for publication

The authors agree to the publication of the manuscript.

## Authors contribution

KCB was the managing physician**,** JO initiated and assessed the CTs of the lung JB accompanied and stimulated the examination, TZ worked on the manuscript.

## Ethics statement

Approved by ethic committee of Charité (No. EA 1/098/18). Written informed consent was obtained.

## Funding

Not applicable.

## Availability of materials

Not applicable.

## Declaration of competing interest

The authors state that they have no conflicts of interest (COIs) related to the work. JB has received honoraria from Novartis.
