# Supplementary material for: Successful simultaneous targeting of IgE and IL-5 in a severe asthmatic patient selected for lung transplantation
Source: World Allergy Organ J. 2022 Jul 31;15(8):100669. doi: 10.1016/j.waojou.2022.100669 (PMC9356159; doi:10.1016/j.waojou.2022.100669)
Supplement: Multimedia component 1 [file mmc1.docx]

**List of differential diagnosis worked up in the case**

The following differential diagnoses were considered

1. Allergic asthma bronchiale approved by positive family history, allergic rhinoconjunctivitis caused by pollen and animal dander, subsequent occurrence of asthmatic symptoms since early childhood, IgE-specific antibodies in the blood, positive methacholine test.
2. Bronchiectasis approved by bronchoscopy and computer tomography
3. Interstitial lung disease ruled out by multiple computed tomography scans of the thorax.
4. Bronchial tree abnormalities not seen in bronchoscopy
5. Cystic fibrosis ruled out at childhood by a negative sweat test
6. Neuromuscular diseases ruled out by genetic tests

- type III spinal muscular atrophy

- type II glycogenosis

7. Pulmonary hypertension excluded by echocardiography and thorax CT
